# Controlled Information Transfer Through An *In Vivo* Nervous System

**DOI:** 10.1038/s41598-018-20725-2

**Published:** 2018-02-02

**Authors:** Naveed A. Abbasi, Dilan Lafci, Ozgur B. Akan

**Affiliations:** 1 Next-generation and Wireless Communications Laboratory (NWCL), Department of Electrical and Electronics Engineering, Koc University, Istanbul, 34450 Turkey; 2 Internet of Everything Group, Electrical Engineering Division, Department of Engineering, University of Cambridge, Cambridge, UK CB3 0FA

## Abstract

The nervous system holds a central position among the major in-body networks. It comprises of cells known as neurons that are responsible to carry messages between different parts of the body and make decisions based on those messages. In this work, further to the extensive theoretical studies, we demonstrate the first controlled information transfer through an *in vivo* nervous system by modulating digital data from macro-scale devices onto the nervous system of common earthworms and conducting successful transmissions. The results and analysis of our experiments provide a method to model networks of neurons, calculate the channel propagation delay, create their simulation models, indicate optimum parameters such as frequency, amplitude and modulation schemes for such networks, and identify average nerve spikes per input pulse as the nervous information coding scheme. Future studies on neuron characterization and artificial neurons may benefit from the results of our work.

## Introduction

With steady miniaturization of scientific applications over the last century, we are at the dawn of nanotechnology. Many interesting applications of nanotechnology such as nano-switches, actuators, intelligent drug delivery systems, nano-scale sensing and bio-hybrid systems are being proposed and realized^1^. Most nanotechnology applications require several nanomachines to operate in synchrony among themselves or with other devices. Therefore, nanomachines require communication with other nanomachines and macro-scale devices. However, there are number of constraints in terms of power, processing, limited memory resources and simple networking that limit the capabilities of nanomachines.

Many paradigms such as nanomechanical, acoustic, electromagnetic, chemical and molecular communications are suggested for nanonetworks, however, the most promising one is molecular communication, where molecules are used to encode, transmit and receive information. Having optimized over millions of years of evolution, these networks are found all around and within us. Molecular communication has the added advantage of being bio-compatible with lower power requirements, thus making it superior to other nanonetworking techniques^2^.

Big networks in living organisms such as human beings communicate among themselves and on macroscopic scale based on molecular communications, on the cellular and intercellular level^3,4^. Understanding the dynamics of molecular communication in living entities not only guides the development of new nanonetworking systems, it also gives new directions to study ourselves and figure out solutions to the problems our bodies face. Many diseases are actually failures of certain types of molecular communications within or among our cells. Hence, by exploring these systems, we take an important step forward.

The nervous system is one of the most important and complex nanonetworks of an animal’s body. This system is based on nerve cells known as *neurons*. Neurons are connected to sensory elements in animal bodies from where they carry information about internal and external stimuli in the form of impulses, known as action potentials or nerve spikes, to the central nervous system (CNS) that is also composed of neurons. The CNS neurons take decisions based on the received impulses and the resulting decisions are then communicated by a separate chain of neurons to muscle cells for further action^5^. Individual neurons or their small networks operate on small information capacity, however, larger networks based on these units such as the human brain are able to achieve the ability to perform very complex decision making tasks.

Many emerging medical applications such as body area networks (BAN) and brain-machine interfaces are targeting wearable electronics that communicate directly with the nervous system^6^. Targeted drug delivery is another important direction where a lot of research is being conducted^7^. Since the nervous system acts as a wired network that is spread throughout the body, it can be used to reliably control various devices connected to the body. Bio-computers based on actual neurons are also envisioned as alternative computing paradigm^8^. Additionally, diseases due to failures of communication in the nervous system such as Alzheimer’s disease, Schizophrenia and Parkinson’s disease are key challenges in the current age^4^. Therefore, the study of communication in the nervous system from the perspective of information and communication technologies (ICT) is an important direction for research efforts.

Several classical models for neurons, their networks and neural coding based on circuit models of neurons are provided in^5^. A number of contrasting theories exist on the information content of nervous signals and neural codes^5,9^. On the other hand, ICT modeling and analysis of the nervous system has only been targeted more recently. A physical channel model for neuro-spike communications in neurons is provided in^10^. Communication-theoretical analysis and models of synaptic multiple-access channels for hippocampal-cortical neurons and cortical neurons is presented in^11^. In^12^, a queueing-theoretical delay model for the nervous system is described. The processing of the presynaptic terminals is discussed in^13^ and the dendritic is described in^14,15^. Despite several communication models, to date there has been no attempt to communicate data through the nervous system. In this regard, we are working towards a unique test bench of the nervous system that achieves data communication through living neurons.

The first step in the development of an experimental data communication system based on the nervous system is the selection of an appropriate neuron type and a testing animal such that the results of such experiments may be extrapolated to other animals. The selected animal should suit the experimental setup and be easily available as well. Keeping these requirements in mind, we see that a vast majority of animals including humans are bilaterians. A fundamental bilaterian body form consists of a tube containing major nerves in a hollow cavity running from the brain to other parts of the body, where each side of the body is identically served by nerves that shoot from the central nerves.

Worms are amongst the simplest bilaterian animals making them a sample bilaterian case^16^. Nerve cords run along the length of a typical worm body merging at the mouth and the tail. The central nerve cords are connected to other nerves like the rungs of a ladder throughout the body. Two ganglial nervous tissues at the head function similar to a simple brain. Worm neurons are covered by sheaths similar to myelin coating of the CNS in higher animals like humans^16^. Although this covering of neurons is not as good as the myelin sheath of human neurons, it offers significant conduction speed in comparison to other invertebrates such as cockroaches, and thus, brings their model system near to that of vertebrates. The size of worm neurons is also large as compared to other invertebrates making them easy to clamp into by means of external probes. Apart from the similar structure, the ease of availability of common worm types and their easy upkeep makes them the ideal candidate for nervous system experiments.

Earthworms are common segmented worms found in soil. The nervous system of an earthworm comprises of a ventral nerve cord and peripheral nerves. Since their neurons are not truly myelinated, the axonal diameters are large which results in giant fibers^16^. They generally have one medial and two lateral giant fibers all being bilateral in conduction^16^. Medial giant fibers respond to stimuli from anterior end and lateral fibers respond to stimulation from the posterior end^17^. Therefore, the median giant fiber channel and the lateral giant fibers channels have two different set of inputs and can be separated spatially by applying inputs to different parts of the earthworm body. The two kinds of fibers are also distinguishable by their conduction velocities. Lateral fibers conduct action potentials slower than the medial fibers^17^.

Extracellular recording of earthworm nervous spike signals by means of external electrodes is easy because of the thin body wall and muscles^18^. The nervous system of earthworms is sensitive to both mechanical and electrical stimuli and responses can be recorded from intact, awake worms where muscle potentials due to body movements are recorded beside action potentials. However, these peripheral sensory cells and muscles can be inactivated with a proper level of anesthesia thereby providing records only from the giant fibers that are still active. Such isolated nervous systems are ideal for experiments with external data signals^18^.

Therefore, having the discussed motivation, we implemented a data communication system between macroscopic instruments and a nervous channel. To achieve this, we first model the nervous channel, and identify the parameters required for this communication system. Data translated as an amplitude and a frequency is then modulated onto the network of neurons to be received at the other end of the nerve cord. The strategies for modulation and symbol assignment are also investigated. The unique contributions of this work can be summarized as follows:

- To the best of our knowledge, this is the first demonstration of a controlled information transfer through an *in vivo* nervous-system-based channel.
- This is the first example of a macro-to nano-scale communication using biological nanonetworks in multicellular animals and among the very few examples of experimental molecular communications^19^. This study will impact not only as a test bench for molecular and nervous communication, it also has the potential to act as a test bench for artificial neurons and synapses.
- The results and analysis of our experiments provide a method to model networks of neurons, identify optimum input parameters such as the frequencies, amplitudes and the modulation schemes. This can pave way for the design, testing and implementation of future nanonetwork applications based on the nervous system.
- We show that the information modulated onto the nervous channel is stored in the number of *nerve spikes per pulse of input signal*.

The remainder of the paper is organized as follows. In Section II, we present the experimental setup and methodology to model and implement the nervous system based communication channel. In Section III, we discuss the results of our experiments. Finally, we conclude the paper in Section IV.

## Methods

Our experiments in this paper can be divided into two categories on the basis of experimental setups. The first setup is used to evaluate the parameters required for the operation of a physical nervous channel, to calculate the channel propagation delay and to model the networks of neurons in this channel. A second similar but more elaborate setup is used for data communication through the nervous channel. The experiments for modeling and data communication are conducted for the medial giant fibers in most cases by stimulating an earthworm at its anterior end and receiving the signals at the posterior end. Similar results are expected for the lateral giant fibers, however, due to their slower conduction speeds and larger activation potentials, lower data rates and higher propagation delays are expected. For each setup, a complete reading from one earthworm is considered if an earthworm gives values for all the test points. Cases where an experiment could not be completed because of nerve damage due to muscle movement are not considered in the results and analysis afterwards. To avoid any bias, readings from the same earthworm are not used twice in the analysis. After giving a brief discussion about the earthworms used in these experiments, we take a look at each of the setups.

### Earthworm Selection and Experimentation

The experiments for this work started with two earthworm species, namely, Lumbricus Terrestris and Eisenia veneta. Both of these earthworms are fairly common and can be found easily in moist soils. Eisenia Veneta are physically bigger and their body wall is stripy as compared to Lumbricus Terrestris^20^. The identification and discrimination of these two species is based on physical features described in^20^ and the operation of CNS for both the worm species is as described in the previous section. Our empirical experiments show that Lumbricus Terrestris exhibit better nervous signal quality because of a thinner muscle layer but they frequently break during stimulation experiments, rendering the data of incomplete sessions useless. Therefore, the experiments reported in this paper are based on our results for the Eisenia Veneta. The words earthworms and worms are used interchangeably hereafter in the manuscript to mean Eisenia Veneta.

Mature worms are chosen intentionally for the experiments because of their larger sizes, where the worm maturity is recognized by the presence of clitellum, which is non-segmented region toward the anterior end of the worm^16^. The worms used for the experiments are kept inside moist soil drums in our laboratory that match their natural habitat. Worms on which an experiment is conducted are subsequently separated from others so that they are not used in an experiment again inadvertently. The worms are anesthetized using an ethyl alcohol solution before the start of an experiment to reduce their movement during experiments, which is a major reason for nerve breakage. Earthworms can regenerate nerve damage and can even grow back their missing tail sections^21^. Therefore, worms are not harmed by the experiments and all worms used in the experiments survived and recovered afterwards.

### Experimental Setup For Modeling of Networks of Neurons

**Figure 1:**
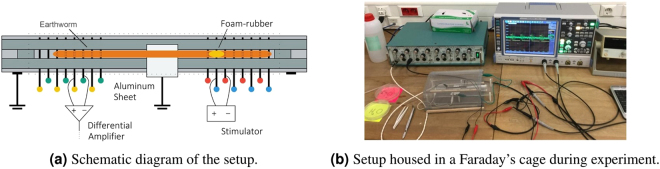
Experimental setup for the modeling of a network of neurons.

To measure the characteristics of the nervous channel and to model the network of neurons present in it, we used the setup detailed in Fig. 1. As discussed earlier, the worms are anesthetized before an experiment using a low percentage alcohol solution (not more than $\mathrm{20 \% }$) to reduce their movement and the associated muscle potentials. During our initial experiments we observed that nervous signals are not recoverable in case an earthworm is moving because muscle potentials, being several orders of magnitude larger than action potential, make the detection of valid nervous spikes very difficult^18^. Although anesthesia affects the nervous system of the worm along with its muscle cells, this trade-off is essential for a meaningful experiment. After being washed and dried, anesthetized worms are placed on the setup as shown in Fig. 1. A grounded aluminum sheet is placed underneath the earthworm to isolate the transmitter and the receiver by ensuring that the stimulator signal does not flow directly through the worm skin or tissues as suggested by^18^. The lack of this unwanted coupling is verified when nervous spikes are received at the receiver instead of the transmitted pulse. Additionally, there is also a delay between the transmitted and received signals.

Pin electrodes may be used to connect the stimulator and the differential amplifier at two points each by either putting the electrodes on the worm skin or by inserting the electrodes inside the earthworm body. The second method is preferred because it keeps the worms firmly in place during experiments and provides better nervous signals because of a higher proximity to the nerve cords. An alcohol solution may be poured over the worm several times during the course of an experiment to keep it anesthetized.

At the start of each experiment, we first test the setup by applying a direct current (DC) signal of appropriate voltage at the stimulator electrodes and observe the corresponding action potentials. Since the nervous spikes of a particular earthworm are the same shape and voltage, the signals thus measured are used as a benchmark for the rest of the experiment. If similar nerves spikes are generated by applying alternating current (AC) waveforms, we proceed with the experiment since it is confirmed that there is no leakage coupling between the input and the output.

#### Stimulator

The stimulator is an isolated unit of electrical stimulation that is capable of producing single or multiple electrical pulses with variable amplitudes, different waveform shapes and stimulus frequencies over the range of at least $1-100Hz$. We use GW Instek SFG-2004 synthesized function generator that provides the required signals for our experiments.

#### Differential Amplifier

Having a myelin like neuron coating offers a challenge since tapping into the nervous signals in such cases requires high quality amplifiers with features such as low noise and high gain. This can be achieved by a number of extracellular amplifiers available in the market. We use AD instruments AM 17 extracellular amplifier to amplify the received signal $1000$ times for our experiments. Since extracellular amplifiers are very sensitive to external noise, the setup is placed inside a Faraday’s cage to cancel external noise sources. The Faraday’s cage is connected to the system ground to provide a common ground connection. If the extracellular amplifier includes filters, they may also be used to condition the signal further. We used filters to cancel high frequency noise form our measurements.

#### Display and Signal Acquisition

The equipment for visualization and recordings of the nervous signal consists of an oscilloscope. We use Rhode and Schwarz RTO1024 digital oscilloscope for data acquisition and storage. The data processing on the recorded signals is further achieved in MATLAB.

### Experimental Setup For Data Communication

**Figure 2:**
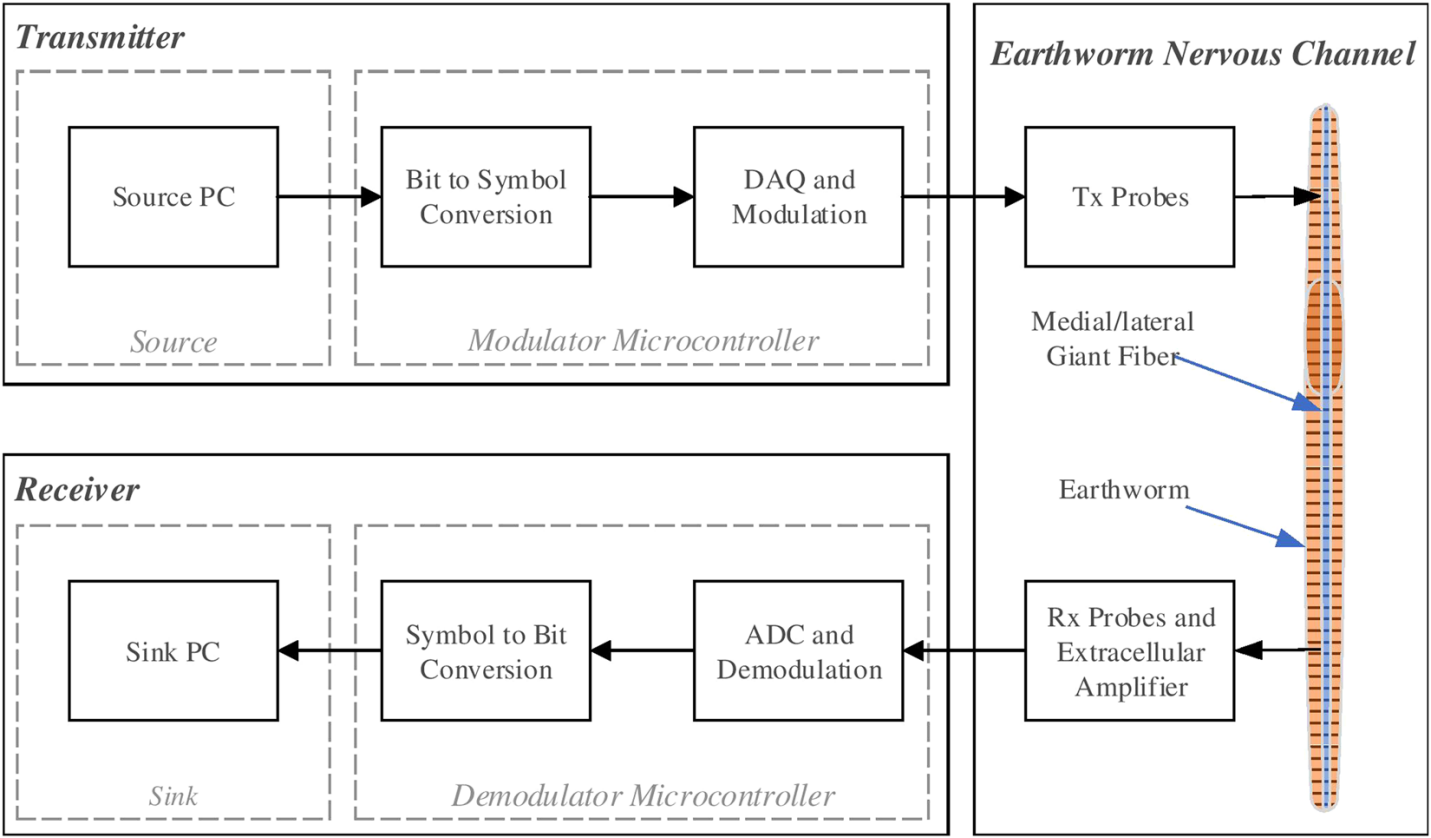
Experimental setup for data communication through the nervous channel.

The channel setup of data communication is similar to the previous setup. At the start of a data transmission, an anesthetized earthworm is placed on the setup shown in Fig. 1 with both the probes of the transmitter (at the stimulator position) and the receiver (at the extracellular amplifier position) inserted inside the worm body. The block diagram for the communication system is given in Fig. 2. We take a look at the major blocks of this setup below.

#### Channel Response Evaluation

Before the start of a transmission session, the transmitter sends a pre-known sweep of different frequency pulses with various amplitudes across the channel and the receiver stores the number of nerve spikes generated by each pulse. This creates an effect similar to channel equalization and helps the receiver to generate an estimate of the channel. We also evaluate the symbol-nerve spike relationship so that the number of nerve spikes received can converted to a valid symbol afterwards. This step is important because there is a trial-to-trial variation in natural systems even between different organisms of the same species.

#### Source

The information to be transmitted through the channel is prepared in the form of a random binary sequence at the transmitter personal computer (PC). To achieve maximum uncertainty, the input sequence is generated such that the zeros and ones are uniformly distributed. Although source encoding is not considered in our current results since we are more interested in the overall channel response to a maximum of uncertainty, schemes such as Lempel-Ziv are applicable to the current scenario and may be investigated in further studies^22^.

#### Modulator

The transmitter PC passes the data bits to a microcontroller that acts as a modulator by means of a serial port (RS232) connection. The directives for the modulation scheme are also sent by the PC to the microcontroller. These directives define the voltage levels and the frequencies that the microcontroller is required to generate for each symbol. Afterwards, the microcontroller breaks the bit sequence into bits required per symbol and transmits each symbol with the specific voltage and frequency for that symbol. Any microcontroller with a digital to analog converter may be used as the modulator. We use Arduino UNO to generate pulses of various frequencies and duty cycles. To vary the amplitude of the signal, external resistors are used. Such a modulator is able to perform on-off keying (OOK), amplitude shift keying (ASK) and frequency shift keying (FSK) in the current system. We discuss these in detail later on in the text.

#### Transmission and Detection

The pulses of each symbol are applied by the stimulator probes. The sequence of action potentials generated as a result of the transmission signal then travels through the worm to be received by the receiver probes where amplification is necessary since the signals are on the orders of a few millivolts. An extracellular amplifier takes the input from the receiver probes and passes them to the demodulator microcontroller after amplification.

#### Demodulator

The demodulator microcontroller works as a pulse counter and counts the nerve spike pulses in an interval equal to the interval for the highest symbol frequency on one of its analog input ports. Our experiments show that for all cases the nerve spikes of the received signal can be bunched together in half of the symbol interval for this frequency. This ensures that different frequency symbols that are sent one after the other from the transmitter are reliably received. Based on the equalization data received at the start, the number of pulses is then converted to a symbol. The demodulator is also arduino UNO based, which is connected to the receiver computer through a serial port.

#### Sink

A PC acts as a sink where post transmission analysis such as visualization and bit error rate (BER) calculations can be conducted.

## Results and Discussions

### Parameter Optimization

The first step in preparing the grounds for data communication is the estimation of experimental parameters that provide an ideal scenario for spike generation and transmission. These parameters include the time for anesthesia, the voltage levels necessary to generate spike signals and the type of transmission waveform. Knowing these details and selecting the best parameters ensures that the physical layer of the nervous channel operates effectively throughout the experiments. All the results of the section are averaged over $30$ different specimens. To make the analysis more straightforward, solid lines in the results of this section are fitted over data points using smoothing spline fitting to show trends.

#### Anesthesia Time

**Figure 3:**
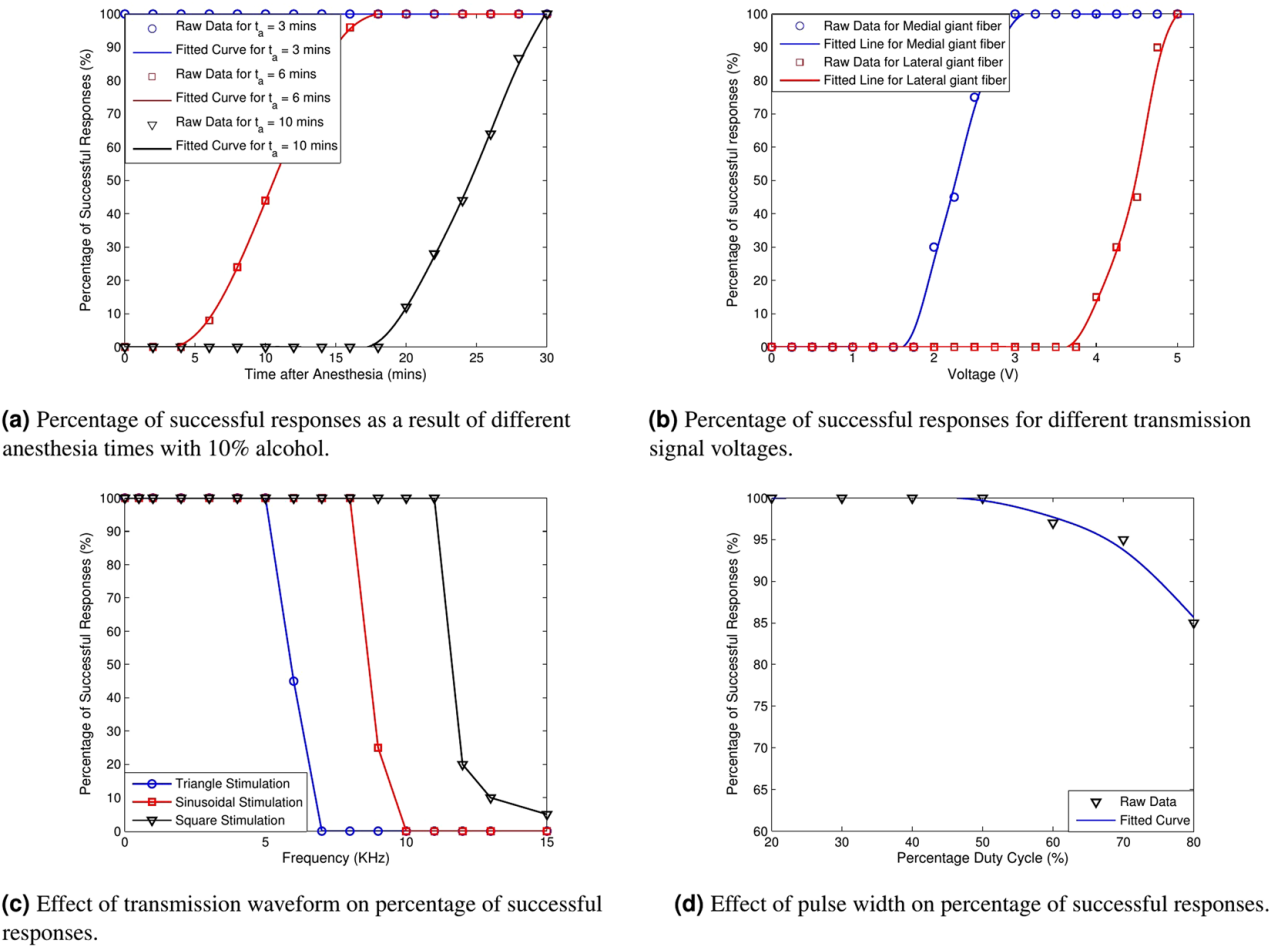
Optimization of experimental parameters.

Our first experiment focuses on determining the appropriate amount of anesthesia required to achieve high reliability signals from a worm. Each reading for this experiment is taken $2\,minutes$ apart with electrical stimulation using a DC voltage of $3V$ that is applied momentarily on the stimulator while keeping the distance between the stimulator probes as $1cm$ and the percentage of successful responses is noted. The percentage of successful responses denotes the percentage of cases where an input signal successfully elicits nerve spike signals from the nervous channel. The results of the experiment are shown in Fig. 3(a) for various anesthesia times, ${t}_{a}$. We see that with a solution of $\mathrm{10 \% }$ alcohol and $3\,minutes$ of anesthesia, the earthworms are ready for the experiment right away. With longer times in anesthesia or higher concentrations of alcohol, the worms take considerable time to be ready for the experiment. Higher concentrations of alcohol are not recommended since the process damages the worms irreversibly and they may even die. Since the process of anesthesia depends on the health, age and type of worms, a small trail-to-trial variation may be expected in the results.

#### Transmission Signal Level

The optimal voltage level of the transmission signal to create action potentials is a very important parameter for the data communication experiment to operate properly. For this experiment, we sweep a DC voltage in the range of $0\mbox{--}5\,V$ with a resolution of $0.25V$ and average our results for both the medial and the lateral giant fibers to generate Fig. 3(b). With the distance between the stimulating electrodes fixed at $0.5cm$, momentary pulses ($0.5\,seconds$ and $10\,seconds$ apart) of DC voltage are given to the earthworm. The results show that action potentials are easily generated on a smaller voltage levels in the medial giant fiber, which is thicker than the lateral giant fibers. For the medial giant fiber, a voltage above $3V$ DC is necessary for reliable transmission of the signal whereas for the lateral giant fibers, a voltage of at least $4.5V$ is necessary.

#### Transmission Waveform

The transmission signal waveform is very essential to identify an appropriate carrier signal that can reliably transmit data over the nervous channel. In this set of experiments, three different types of waveforms, namely, the square wave, the sinusoidal wave and the triangular wave, are applied to the medial giant fiber at different frequencies and the percentage of successful responses from the receiver are noted. The peak-to-peak voltage values were set at $3.5V$, which is usually enough to generate action potentials. Each waveform is applied to the anterior end of the worm for $0.5\,seconds$ and the corresponding action potential generation is noted. The results are shown in Fig. 3(c).

The results show that at lower frequencies, all types of transmission waveforms perform very reliably, however, once the frequency increases, the square wave results in most successful generation of action potentials on application of short pulse, followed by the sinusoidal and the triangular wave. The reason for this lies in the fact that amongst the waves applied, square wave has the highest root mean square (RMS) voltage and the effective current through the worm body to create transmission pulses decreases with frequency. Therefore, square pulse based transmission signals provide the best results on the nervous channel. Since the waveform analysis is independent of the type of neuron, a similar result is expected for the lateral giant nerves.

#### Transmission Pulse Width

Now that the square pulse is identified for generation of transmission pulses, pulse width becomes an important parameter. The minimum pulse width that can generate an impulse should be used to reduce the power requirements of the system while ensuring minimum damage to the worms. In this regard, by using signal frequency of $10Hz$, signals of different pulse widths were applied to the transmission end of the medial giant fiber and the resultant percentage successful responses are shown in Fig. 3(d).

The results show that with a pulse width with $\mathrm{20 \% }$ on-time, very reliable communication can be achieved. Since this reduces the power requirements of the transmitter as well as any potential damage to the worm, it is selected for subsequent experiments.

### Channel Propagation Delay

The overall channel propagation delay of a network of neurons depends on the axonal conduction velocity, dendridic conduction delays and diffusive delays along the length of the network^12^. Since diffusive delays cannot be singled out without the knowledge of the exact physiology, they are considered as a part of the channel conduction velocity. Additionally, it should be noted that diffusive delays are very small in comparison to axonal transmission delays that may be neglected altogether for neurons of sufficient length^12^. The overall channel propagation delay for a network of linearly connected neurons is^12^

1 $$R=\frac{L}{{v}_{avg}},$$

where *L* is the length of the channel and *v*_*avg*_ is the measured conduction velocity. Our results show that the average conduction velocity for the medial giant fibers is $13.196\,m/s$ and $\mathrm{5.48\ }m/s$ for the lateral giant fibers. These agree with previous studies and show that the lateral medial fibers have higher propagation delays^17^.

### Modeling of Networks of Neurons

In order for a communication system to be established, we should know how the channel translates an input to an output. Certainly, a neural coding mechanism is converting the information we provide to the channel to nervous spikes that then travel through the network. Therefore, the most important question is how information is modulated onto the channel as a result of different inputs. A number of neural coding theories try to answer this question by describing information content of nervous signals by various rate metrics^5^. However, the exact mechanism for our scenario can only be understood once a system model is developed.

The system model may be based on available ICT studies^10–15^, however, these either target constituents of a single neuron or extend only to a small number of neurons. Additionally, the nervous system has an interdependency on a number of other body networks such as the insulin-glucose system^23^ and for a scenario where both the nervous connections and the constituent systems are unknown, such methods are not applicable.

Recently, OpenWorm was started as an open-source initiative to model the complete body of small unsegmented nematodes, Caenorhabditis elegans (C. elegans)^24,25^. As part of the model, OpenWorm provides the ‘connectome’ for C. elegans which is basically a map of all the neurons of the nervous system and their connections. The model also provides muscular, soft tissue and fluid models for the organism. Such a detailed model is quite viable to perform the task of modeling communication through the nervous system, however, it is not applicable to the current study because both earthworms and C. elegans belong to different phyla and thus, have quite disparate body structures. Additionally, an earthworm’s nervous structure is much larger than the nervous system of a C. elegans. Therefore, a different approach is required for the current task.

**Figure 4:**
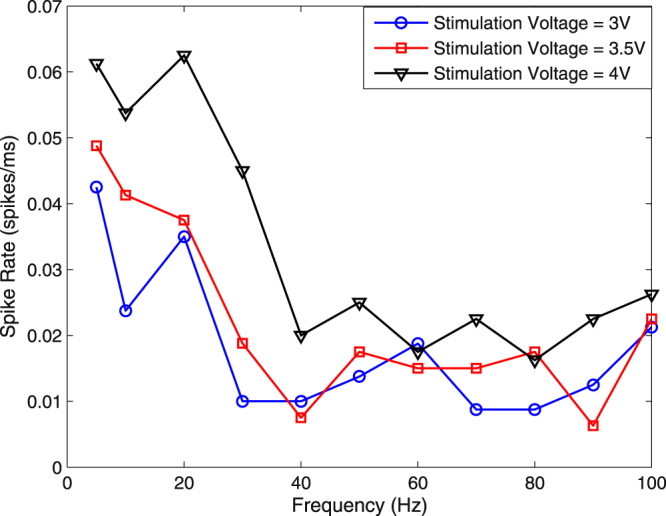
Effect of frequency on average spike rate.

To model the network of neurons present in the nervous channel of an earthworm, we consider it as a black-box and analyze the inputs and outputs of the system to find a relationship between them. Black box modeling can be efficiently conducted for the analysis of a network of neurons because neurons respond to a limited range of inputs that may easily be replicated under laboratory conditions. To achieve the black-box modeling, we sweep signals with various frequencies and amplitudes in an orderly manner by applying each frequency and amplitude signal for at least $10$ seconds as the input of the nervous channel and record the outputs from the extracellular amplifier. A square pulse with a duty cycle of $\mathrm{20 \% }$ is used to this end. The ordering of the tests is shuffled to prevent any biases in the data. We tested several schemes of neural coding to evaluate which among them best describes the information we pass onto the channel. These included average spike rate, instantaneous spike rates, spike width analysis and inter-spike delay. However, none of these metrics truly represents the information we modulate. For instance, Fig. 4 shows effects of changing input voltage and frequency on the average spike rate. We see that the spike rate does decrease in general as the frequency increases, however, the relationship is not straight forward and at multiple points, the data does not describe the unique input we give. The same holds for an instantaneous spike rate case. Further, our analysis reveals that there is no information in the inter-spike delays that can be estimated as a log-normal distribution with a mean of $1ms$ and a standard deviation of $0.1\,ms$. The spike width also stays nearly unchanged over a specimen having mean values of around $2\,ms$.

**Figure 5:**
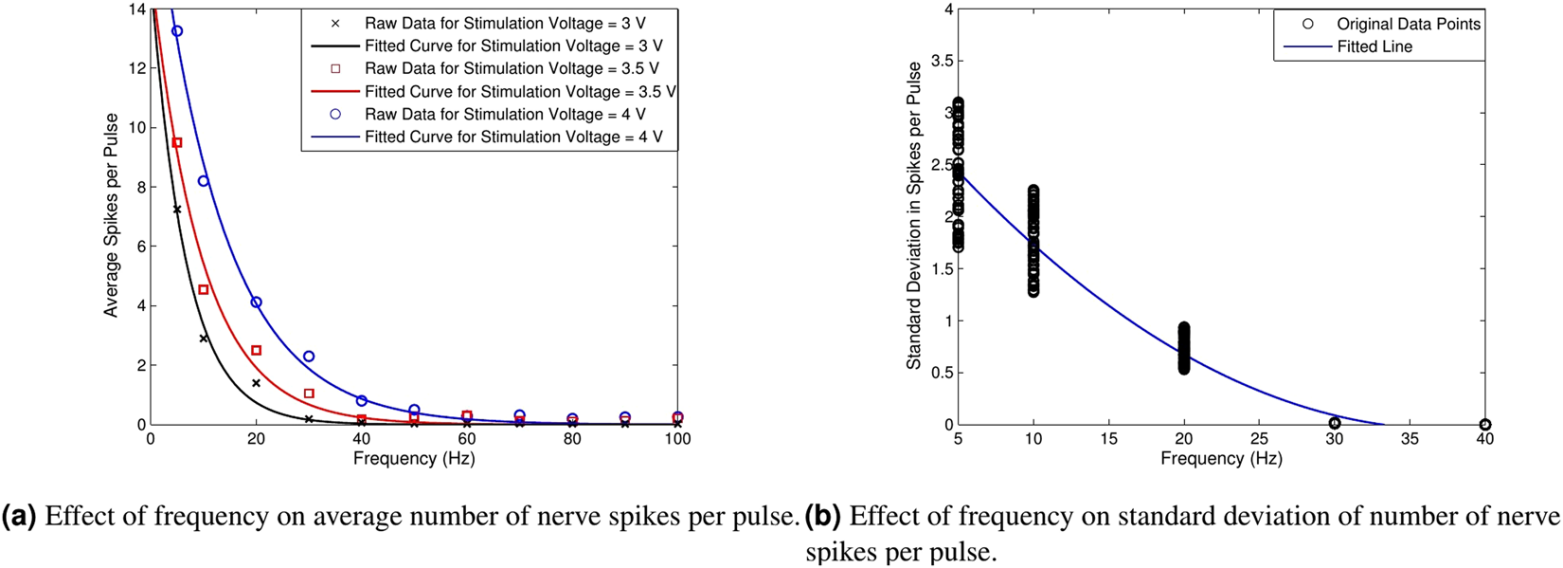
Analysis for number of spikes per input pulse.

We then tested *nerve spikes per input pulse* as a measure of the information content. The results of these experiments for $50$ worms are summarized in Fig. 5. We see that the resultant data describes the information we gave to the channel in an orderly fashion. Figure 5(a) shows how the number of nerve spikes per input pulse reduce as the frequency increases. Using an exponential function, we fit the data points by minimizing the sum of squared errors (SSE) as

2 $$Y(x)=a{e}^{-Xb},$$

where $Y$ represents the number of nerve spikes per pulse generated by a frequency $X$, while $a$ and $b$ are constants that change from worm to worm. Our experiments show that data from each individual earthworm can be fitted using this function with different values of the constants $a$ and $b$. The rationale behind suggestion of an equalization block in the data communication is to communicate the values of these parameters to the receiver for a particular earthworm. The number of nerve spikes per pulse varies from its mean value randomly with a standard deviation term $\sigma$ shown in Fig. 5(b). We can observe that for lower frequencies, where the number of nerve spikes per pulses may be higher, $\sigma$ is higher. $\sigma$ is modeled by minimizing SSE for a second degree polynomial function as

3 $$\sigma (x)={p}_{1}{x}^{2}+{p}_{2}x+{p}_{3},$$

where the average values of the constants are found to be ${p}_{1}\,=\,2.34\times {10}^{-3}$, ${p}_{2}=-1.756\times {10}^{-1}$ and ${p}_{3}\,=\,3.252$. Thus, the network of neurons for earthworm medial giant can be modeled by using (2) and (3). This black-box modeling approach can be used with any network of neurons available in an experiment.

### Data Communication through Networks of Neurons

From the results of Fig. 5(a), we see that a number of modulation schemes can be employed for data communication through the nervous system of an earthworm. We may employ on-off keying (OOK), amplitude shift keying (ASK) or frequency shift keying (FSK). For instance, for OOK we can use any frequency in the response range of the network with an amplitude above $3\,V$ and select the absence of a signal as the off case. Similarly, for ASK, we may select a particular frequency for the modulating signal and send different amplitudes as different symbols. This is especially true for lower frequencies, where the difference of pulses between two amplitudes is higher. Similarly, keeping a constant amplitude, different frequencies may be used to generate the symbols for FSK. ASK operates at lower data rates than FSK, because it provides comparable performance at lower frequencies and may not be feasible at higher frequencies. Taking FSK based on square waves as a sample case of modulation, we perform our analysis in the rest of the section accordingly.

#### Simulation Model

In order to analyze, compare and improve the performance of a communication system, simulations before the actual implementation are standard practice. Thus, we design a simulation of the system that generates $\mathrm{10,000}$ bit randomly that are then modulated using (2) and (3) with log-normally distributed inter-spike delay and a fixed spike width of $2ms$ similar to our finding in the modeling section.

**Figure 6:**
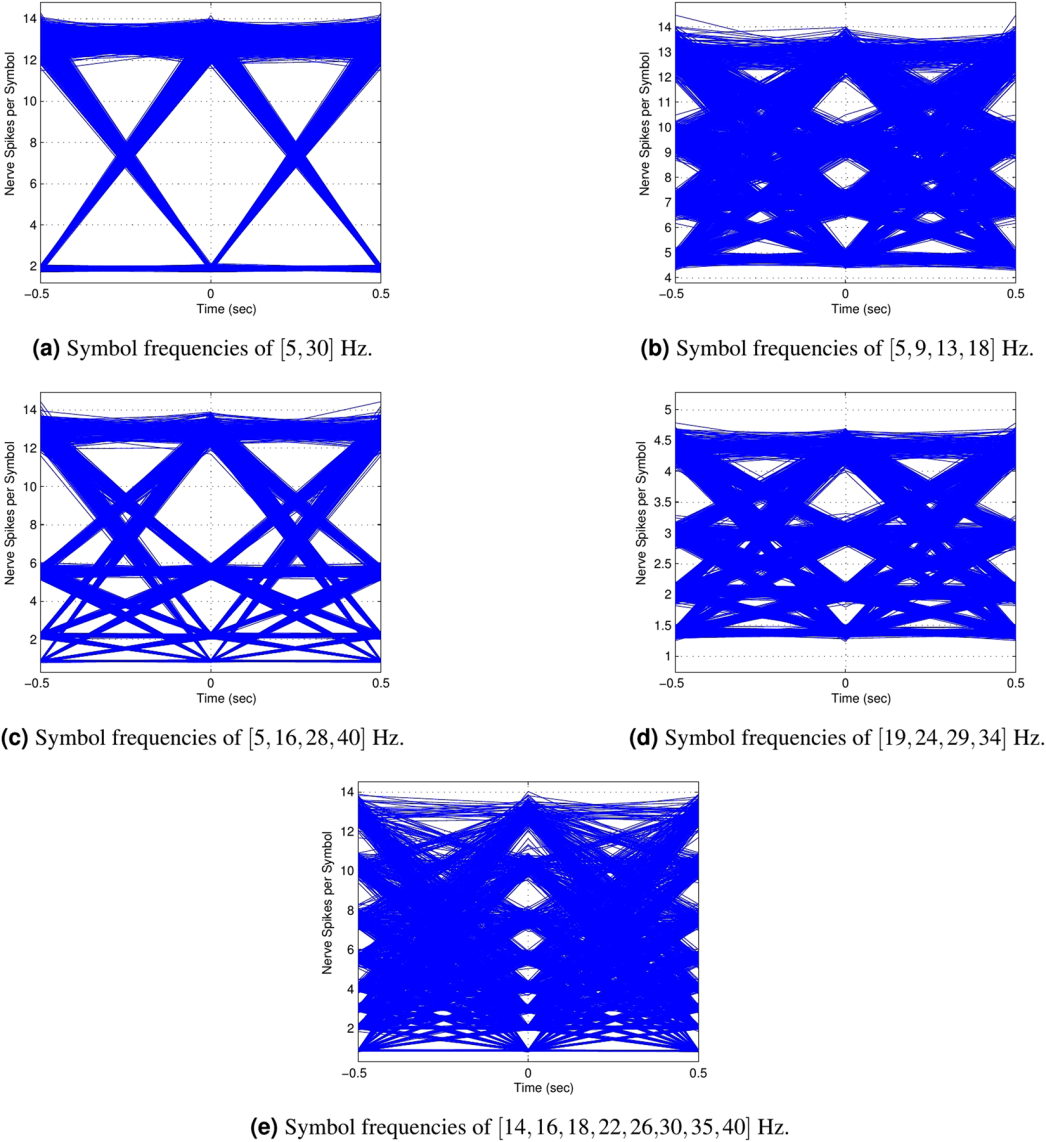
Eye diagrams for FSK with different number of symbols.

To formalize the analysis of simulations, we use eye diagrams on a symbol level. Eye diagrams are used here to identify the average distance between two symbols in terms of nerve pulses generated by each symbol. The diagrams show the amount of distortion present at symbols of different frequencies. This gives us a general idea of expectations from the experiments where eye widths are directly proportional to BER. Although, on simulation level larger eye sizes should correspond to error free transmission, in practice they can only be associated with lower BER. This is because the natural process of nerve transmission can have a large number of random variables such as organism health, exact age, trial-to-trial variability and the environmental conditions of a particular experiment that cannot be quantified in all cases and thus are not considered in the current model. However, the simulations are necessary to identify the optimal symbol frequencies for a particular specimen and also in the identification of decision boundaries for symbols based on the number of spike pulses received. Several eye diagrams generated by the simulation are shown in Fig. 6. These diagrams show how the symbols having different frequencies are translated into number of nerve spikes by the channel. Since the change is governed by a random process, the number of nerve spikes changes around its average value. The opening of an eye in this case shows the margin a receiver has for decisions.

Figure 6(a) shows the results for a modulation scheme using two FSK symbols. We see that the eye opening is very wide and the margins for decision at the receiver are ample. Having a decision boundary at around $7$ pulses, the receiver can easily distinguish between the symbols. We also see that since at lower frequencies the nerve spike number per symbol varies much more, the spikes generated by the $5Hz$ symbol are spread over a large area. In Fig. 6(b–d), we see FSK assignments for four symbols. Clear eye opening are seen in Fig. 6(c) where the symbols are distributed as far as the bandwidth permits. This scheme points to the lowest BER since we cannot misinterpret our symbols. In Fig. 6(b) and (d), the eye openings are much smaller that suggests a higher BER with Fig. 6(d) having a better comparative performance because of slightly bigger eye openings. This is because of the fact that spike variability reduces at higher frequencies. Finally, in Fig. 6(e), we present the case for an eight symbol FSK arrangement. The frequency assignment is done in such a way that the separation at lower frequencies is larger than at higher frequencies. The BER for this case is expected to be higher than all other cases.

## Experimental Results

**Figure 7:**
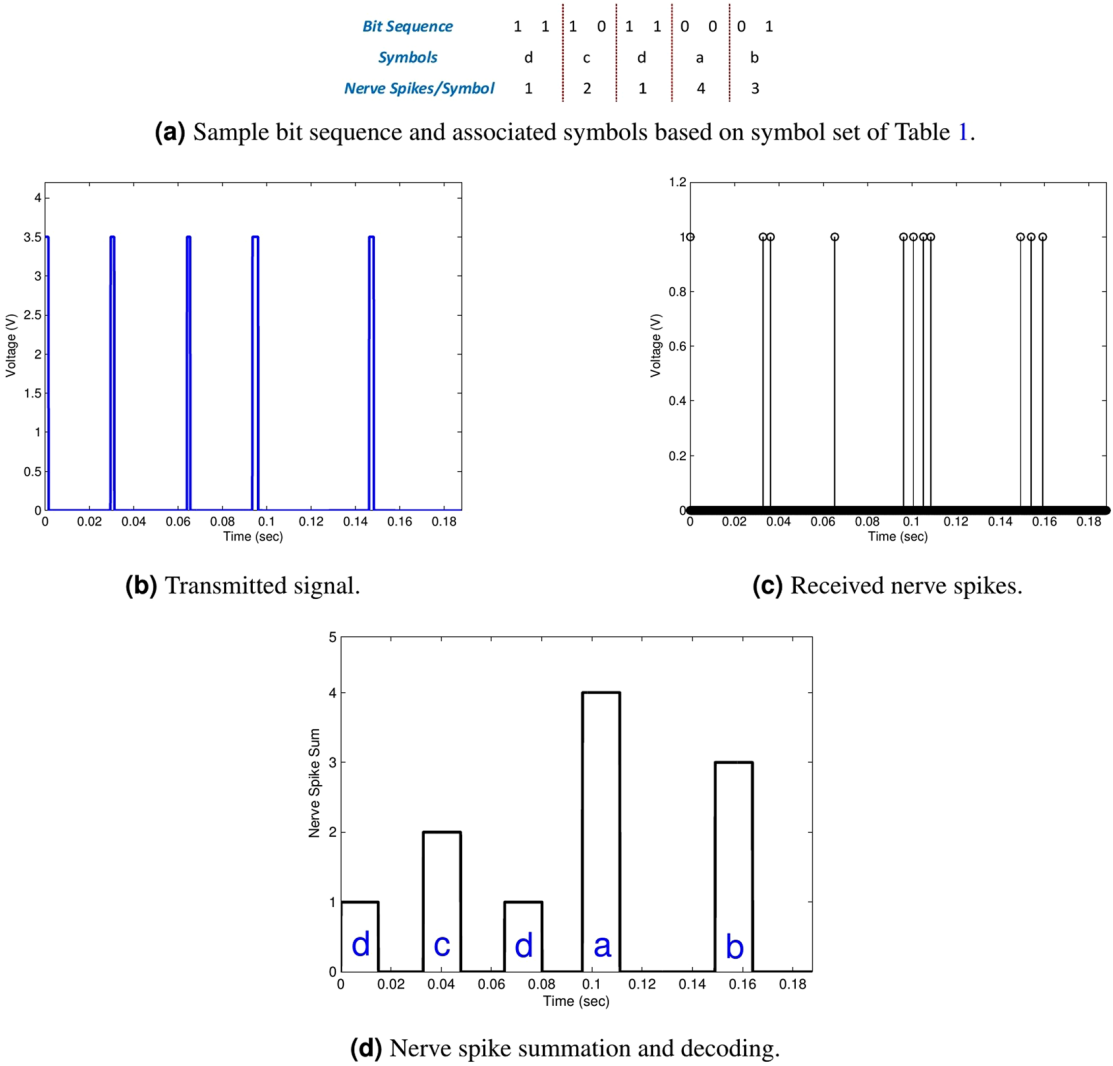
Transmission of a bit sequence over the nervous channel.

After completing the simulation modeling, we perform experiments of data communication, where $1000$ randomly generated bits of data passed through an earthworm. It is ensured that all symbols are equally represented in the transmission data. We first discuss the results of a small binary sequence from one of our data transmissions to describe the communication process in detail. Using known signals for equalization, we measure the values of $a$ and $b$ in (2) as $19$ and $-0.07706$, respectively for one particular worm. The symbol set described in Table 1 is then based on these parameter values. Figure 7(a) shows a small portion of our transmission bit stream comprising of $10$ bits. The transmitter first converts the bits into symbols based on Table 1 and generates the respective frequencies. The transmission signal is shown in Fig. 7(b). The signal is received on the receiver side after an amplification by the extracellular amplifier. After smoothing to remove noise, the received signal is converted to neural spikes in the form of impulses as shown in Fig. 7(c). The pulses are then processed by their summation in windows equivalent to the period of the highest frequency and the resultant signal is shown in Fig. 7(d). Finally, the receiver demodulates the received number of nerve spikes to the nearest symbol. Decoded symbols are overlayed on Fig. 7(d).

**Table 1:** Symbol table for [19, 24, 29, 34] Hz symbol set.

*(Symbol)*	*(Binary Code)*	*(Frequency(Hz))*	*(Average Nerve Spikes/Symbol)*
*a*	$00$	$19$	$4$
*b*	$01$	$24$	$3$
*c*	$10$	$29$	$2$
*d*	$11$	$34$	$1$

The averaged results for $50$ earthworms are shown in Table 2. We see that, as expected, when we use symbol sets that have higher frequencies (Case 3), the BERs are lower compared to symbol sets with lower frequencies (Case 2). This is because of smaller variability in spike number at higher frequencies as shown by Fig. 5(b). Lowest BERs are possible when the symbol set is distributed such that the symbols are at a maximum distance from each other (Case 1). However, we also notice that the best data rates are achieved when symbol sets of higher frequencies are used (Case 3). A result for FSK with eight symbols is also shown (Case 4). We see that the data rate is further increased in this case, however, the BER is the highest. Thus, the results of our implementation of data communication through the nervous system agree with our simulation expectations for BER.

**Table 2:** BER and data rates for various symbol sets.

($\#$)	*(Symbols)*	*(BER)*	*(Data Rate (bps))*
1	$\mathrm{[5,}\,\mathrm{16,}\,\mathrm{28,}\,\mathrm{40]}$ Hz	$2\times {10}^{-5}$	$24.7514$
2	$\mathrm{[5,}\,\mathrm{9,}\,\mathrm{13,}\,\mathrm{18]}$ Hz	$4.6\times {10}^{-4}$	$18.0346$
3	$\mathrm{[19,}\,\mathrm{24,}\,\mathrm{29,}\,\mathrm{34]}$ Hz	$7.2\times {10}^{-4}$	$52.6646$
4	$\mathrm{[14,}\,\mathrm{16,}\,\mathrm{18,}\,\mathrm{22,}\,\mathrm{26,}\,\mathrm{30,}\,\mathrm{35,}\,\mathrm{40]}$ Hz	$6.8\times {10}^{-3}$	$66.6102$

Further improvements may be achieved by taking advantage of source encoding schemes that assign symbols with lower probability to lower frequencies because they present higher variability and reduce the over all data rate. Such improvements may increase the data rate and improve the BER performance further. Since the worms’ nerves run in two directions, with different points of stimulation, a study on bi-directional communication through the nervous system of earthworms can be an interesting future work.

The data rates for a nervous system based data communication system may seem nominal in comparison with contemporary communication system, however, we should note that for animals like humans, higher information processing data rates exist due to naturally existing diversity. Organs like the human brain have billions of neurons working in parallel and such system, therefore, have much higher information capacity than many contemporary systems although their fundamental units have similar performance. Additionally, applications such as BANs that may target the data integration of the nervous system, similar data rates in the human body may be sufficient to transmit and receive sensors data reliably. For similar systems and applications in the human body, stimulation voltages can be calculated in a likewise manner (or taken from existing literature) while the stimulator can be strategically placed in certain regions of the body where nerves are near the skin. Alternatively, electrodes can also be surgically placed in the vicinity of the target neurons as is the case for recent brain-machine interfaces proposals^26^.

## Conclusion

In this work, we realized the first data transfer through an *in vivo* nervous system used as a communication channel on nano-scale. In our system, macro-scale devices are practically connected by the communication channel existing in the nervous system of earthworms. We model networks of neurons by considering them as a black-box and derive the relationship between their input and output to establish average nerve spikes per input pulse as the nervous coding scheme. We further, calculate the channel propagation delay of this network and indicate optimum physical layer parameters such as frequency, amplitude and modulation schemes for such networks. The use of eye diagrams for system evaluation is discussed for simulations of these channels and our results for the physical communication agree with the simulation models. We believe that the experimental results obtained in this work are very encouraging for researchers to develop novel applications based on information exchange with the nervous system. The experimental setup is quite easy-to-implement as an educational tool, a model for biological communication systems and may be used as a test bench for future applications in the nervous system. The study of bi-directional channels and effects of coding schemes are left as future studies. Development of connectomes similar to OpenWorm^25^ and the extension ICT studies to such networks are also an important direction for further research.
